# Feasibility and outcomes of fetoscopic endoluminal tracheal occlusion for severe congenital diaphragmatic hernia: A Japanese experience

**DOI:** 10.1111/jog.14504

**Published:** 2020-09-28

**Authors:** Seiji Wada, Katsusuke Ozawa, Rika Sugibayashi, Fumio Suyama, Shoichiro Amari, Yushi Ito, Yutaka Kanamori, Hiroomi Okuyama, Noriaki Usui, Jun Sasahara, Tomomi Kotani, Masahiro Hayakawa, Kiyoko Kato, Tomoaki Taguchi, Masayuki Endo, Haruhiko Sago

**Affiliations:** ^1^ Center for Maternal‐Fetal, Neonatal and Reproductive Medicine National Center for Child Health and Development Tokyo Japan; ^2^ Division of Surgery, Department of Surgical Specialties National Center for Child Health and Development Tokyo Japan; ^3^ Department of Pediatric Surgery Osaka University Graduate School of Medicine Suita Japan; ^4^ Department of Pediatric Surgery Osaka Women's and Children's Hospital Izumi Japan; ^5^ Department of Maternal Fetal Medicine Osaka Women's and Children's Hospital Izumi Japan; ^6^ Department of Obstetrics and Gynecology Nagoya University Graduate School of Medicine Nagoya Japan; ^7^ Division of Neonatology, Center for Maternal‐Neonatal Care Nagoya University Hospital Nagoya Japan; ^8^ Department of Obstetrics and Gynecology Kyushu University School of Medicine Fukuoka Japan; ^9^ Department of Pediatric Surgery Kyushu University School of Medicine Fukuoka Japan; ^10^ Department of Obstetrics and Gynecology Osaka University Graduate School of Medicine Suita Japan

**Keywords:** adverse events, congenital diaphragmatic hernia, fetal mortality, fetal therapy, fetoscopy

## Abstract

**Aim:**

To present the feasibility, safety and outcomes of fetoscopic endoluminal tracheal occlusion (FETO) for the treatment of severe congenital diaphragmatic hernia (CDH).

**Methods:**

This was a single‐arm clinical trial of FETO for isolated left‐sided CDH with liver herniation and Kitano Grade 3 stomach position (>50% stomach herniation into the right chest). FETO was performed at 27–29 weeks of gestation for cases with observed/expected lung to head ratio (o/e LHR) <25% and at 30–31 weeks for cases with o/e LHR ≥25%.

**Results:**

Eleven cases were enrolled between March 2014 and March 2016, and balloon insertion was successful in all cases. The median o/e LHR at entry was 27% (range, 20–33%). The median gestational age at FETO was 30.9 (range, 27.1–31.7) weeks. There were no severe maternal adverse events. One fetus died unexpectedly at 33 weeks of gestation due to cord strangulation by the detached amniotic membrane. There were 3 cases (27%) of preterm premature rupture of membranes. In all 10 cases, balloon removal at 34–35 weeks of gestation was successful. The median gestational age at delivery was 36.5 (range, 34.2–38.3) weeks. The median duration of occlusion and the median interval between balloon insertion and delivery were 26 days (range: 17–49 days) and 43 days (range, 21–66 days), respectively. Both the survival rate at 90 days of age and the rate of survival to discharge were 45% (5/11).

**Conclusion:**

The FETO is feasible without maternal morbidity in Japan and could be offered to women whose fetuses show severe isolated left‐sided CDH to accelerate fetal lung growth.

## Introduction

Congenital diaphragmatic hernia (CDH), a sporadic defect in the diaphragm, is characterized by visceral herniation into the chest. Advances in ultrasonography in prenatal care have facilitated the prenatal diagnosis of CDH.^1^ Prenatal detection, which allows referral to experienced tertiary centers, and advances in postnatal care for CDH, including gentle ventilation and circulatory stabilization, have improved the prognoses of fetuses with CDH.^2^, ^3^, ^4^ The survival rate of isolated prenatally diagnosed CDH was 79% in a Japanese multicenter study, and subsequent morbidity remained high.^5^ Furthermore, fetuses with heavy visceral herniation, characterized by liver herniation, right‐shifted stomach herniation and small lung size, still have high rates of mortality and morbidity.^6^, ^7^, ^8^ The outcomes of infants with isolated CDH depend on the severity of pulmonary hypoplasia and hypertension, which are derived from lung development *in utero*.^6^, ^9^ The limitations of postnatal care for severe CDH has raised fetal intervention.

The rationale for fetal therapy for CDH is to improve the growth of the hypoplastic lung before birth. After attempting some strategies,^10^, ^11^ fetal tracheal occlusion with a detachable silicon balloon was developed.^12^ The initial randomized study by Harrison *et al*., which used a 5‐mm uterine port with laparotomy, failed to show the superiority of fetal intervention due to a high rate of preterm premature rupture of membranes (pPROM) and preterm delivery.^13^ In 2004, Deprest *et al*. introduced fetoscopic endoluminal tracheal occlusion (FETO) as a minimally invasive procedure performed through a percutaneous approach with a 3.3‐mm cannula.^14^ It is suggested that FETO may improve postnatal survival in fetuses with severe CDH.^14^, ^15^ Several small studies of each institutional experience of FETO have been reported as additional supportive evidence.^16^, ^17^, ^18^, ^19^

In 2014, we started a FETO program that was the first trial of FETO in Asia. The aim of the present study was to report the feasibility, safety and outcomes of FETO for the treatment of severe CDH at our center.

## Methods

This study was a single‐arm clinical trial registered as UMIN000012395 (http://www.umin.ac.jp). Women carrying fetuses with left‐sided CDH at 26–29 weeks of gestation were evaluated at the National Center for Child Health and Development (NCCHD) in Tokyo, Japan prior to participation in the trial. Entry evaluations included detailed ultrasonography, advanced echocardiography and a karyotype analysis by amniocentesis. The stomach position was determined according to Kitano's grading scale.^8^ The observed to expected lung area to head circumference (o/e LHR) was obtained using the tracing method.^7^ Magnetic resonance imaging (MRI) was also performed. Patients were scheduled to undergo FETO at the NCCHD in Tokyo. The protocol was approved by the institutional ethics board (No. 507), and the patients provided their written informed consent.

The eligibility criteria were singleton pregnancy, fetus with left‐sided CDH, liver herniation into the chest, Kitano Grade 3 stomach position (>50% of the stomach herniated into the right chest),^8^ observed to expected lung area to head circumference (o/e LHR)^7^ <45%, no ultrasonographic findings of other anomalies, a normal karyotype, cervical length > 20 mm and no hypertensive disorders. Families of women who met the criteria were extensively counseled by fetal therapy teams of the NCCHD. The timing of FETO differed according to the o/e LHR. FETO was performed at 27–29 weeks of gestation for cases with o/e LHR <25% and at 30–31 weeks of gestation for cases with o/e LHR 25–44%.

The FETO was performed under maternal spinal and epidural anesthesia. Fetal anesthesia and immobilization were performed by percutaneous ultrasound‐guided intramuscular injection of fentanyl (0.01–0.02 mg/kg), atropine (0.01 mg/kg) and vecuronium (0.2 mg/kg). FETO was performed in a procedure similar to that performed in the clinical research consortium.^20^ After selecting the best site of entry, a disposable flexible cannula (RCF 11.0 Check‐Flo introducer sets, Cook) with a pyramidal trocar (11650TG, Karl Storz) was percutaneously inserted into the amniotic cavity under ultrasound guidance. External cephalic version and/or amnioinfusion were added to select the best entry site if necessary. A 1.3 mm semi‐rigid fetoscope (11540AA; Karl Storz) within a 3.3‐mm curved sheath (11540KE; Karl Storz) with a channel for inserting a balloon using a delivery catheter (Bal‐tacci‐BDPE‐100 0.9 mm; Balt) was inserted through the cannula. After inserting the fetoscope into the oral cavity of the fetus, the epiglottis and vocal cords were observed. The fetoscope was inserted into the trachea through the vocal cords, a detachable balloon (Goldbal 2; Balt) was delivered and placed in the middle of the trachea between the carina and the vocal cords with the infusion of 0.6 mL of normal saline solution.

Perioperative management with prophylactic tocolysis and antibiotics and postoperative management until balloon removal were provided at the NCCHD. Patients were followed up with ultrasonography every week to evaluate the balloon location, fetal growth, fetal lung size and amniotic fluid volume. Patients were also followed up with electric fetal monitoring (EFM) twice a week. MRI was performed before balloon removal at 33–34 weeks of gestation. Balloon removal *in utero* was planned at 34 weeks of gestation at the NCCHD. Balloon removal was achieved by puncture under ultrasound guidance or using fetoscope (the same as the balloon insertion technique). A 22 gage PTC needle (Hakko Company) was used for ultrasound‐guided puncture. Using a fetoscope, the balloon was punctured by a sharp stylet (11506P; Karl Storz) with the balloon held by grasping forceps (11510C, Karl Storz) and then the balloon was removed with the fetoscope. If these two methods were not applicable due to pPROM or the onset of labor before elective balloon removal, *ex utero* intrapartum treatment (EXIT) was performed to remove the balloon before birth.

After balloon removal, standard prenatal management with a weekly ultrasound examination and planned delivery and standard neonatal management of CDH were provided at the NCCHD or at one of four referring perinatal centers (Osaka University Hospital, Osaka Women's and Children's Hospital, Nagoya University Hospital and Kyushu University Hospital). All neonates were treated by the standardized postnatal managements of each institute, including gentle ventilation with high‐frequency oscillatory ventilation. CDH repair was performed after respiratory and circulatory stabilization.

Data on the prenatal ultrasound findings, MRI findings, FETO and balloon removal procedure findings, adverse events of fetal therapy,^21^ delivery, CDH repair and neonatal/infantile outcomes were recorded. The primary outcomes were the completion of FETO, the occurrence of severe FETO‐related maternal adverse events, and the survival of the fetus.

## Results

A total of 11 cases were enrolled between March 2014 and 2016. The perioperative findings and outcomes of the cases are shown in Table 1. FETO was performed at 27–29 weeks of gestation in 4 cases and at 30–31 weeks of gestation in 7 cases. Balloon insertion was successful in all 11 cases. Five cases required amnioinfusion. In all 10 cases in which balloon removal was required, it was successfully performed at 34–35 weeks of gestation. A fetoscope was used in 6 cases, while 2 cases were performed under ultrasound guidance. In one case (Case 1), balloon puncture was performed under ultrasound guidance on the day after pPROM and the fetus was delivered 4 days later. Two cases with pPROM underwent EXIT due to difficulties in balloon removal under fetoscopy or with ultrasound guidance. In one case (Case 4), balloon puncture was not succeeded under ultrasound guidance at 34 weeks (2 weeks after the occurrence of pPROM). EXIT was performed electively at 35 weeks of gestation. In the other case (Case 8), elective EXIT was performed at 34 weeks of gestation (4 weeks after the occurrence of pPROM).

**Table 1:** Perioperative findings and outcomes of 11 patients who underwent FETO

Patient no.	Pre‐FETO findings		FETO		Adverse events		Unplug		Outcomes		
	o/e LHR (%)	Placental location	GA (weeks)	Amnioinfusion	pPROM (weeks)	Fetal death (weeks)	GA (weeks)	Method	GA at birth (weeks)	Birth weight (g)	Survival at 90 days
1	33	Post	31.6	No	33	No	34.0	US	34.6	2000	Yes
2	27	Post	31.4	No	No	No	34.3	Fetoscopy	36.4	2182	No
3	32	Ant	31.9	Yes	No	No	34.6	Fetoscopy	37.4	2802	Yes
4	23	Ant	28.4	Yes	32	No	35.4	EXIT	35.4	2248	No
5	28	Lat	31.7	No	No	No	34.3	US	38.3	2502	No
6	30	Ant	30.9	Yes	No	33	–	–	–	1960	–
7	27	Ant	30.7	Yes	No	No	34.7	Fetoscopy	35.7	2010	No
8	20	Ant	27.1	Yes	29	No	34.1	EXIT	34.1	2260	Yes
9	21	Lat	27.6	No	No	No	34.0	Fetoscopy	37.0	2570	Yes
10	31	Post	31.1	No	No	No	34.4	Fetoscopy	36.6	2874	Yes
11	20	Post	27.6	No	No	No	34.6	Fetoscopy	37.7	2880	No

Ant, anterior; EXIT, *ex utero* intrapartum treatment; FETO, fetoscopic endoluminal tracheal occlusion; GA, gestational age; Lat, lateral; pPROM, preterm premature rupture of membranes; Post, posterior; US, ultrasound‐guided puncture.

**Figure 1:**
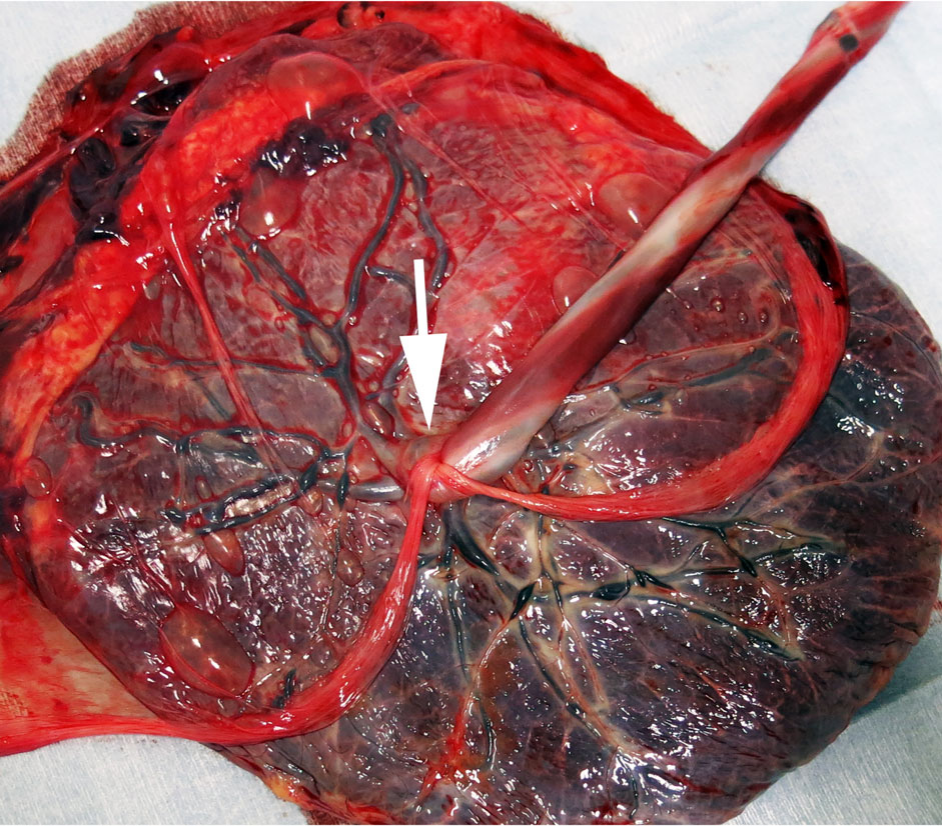
A macroscopic view of the placenta in a case of fetal death (case 6). The arrow indicates cord strangulation by the detached amniotic membrane.

The adverse events of the cases are shown in Table 2. No patients experienced FETO‐related maternal bleeding, placental bleeding or fetal injury. There were no cases with uterine infection, placental abruption or maternal death. One fetus died unexpectedly at 33 weeks of gestation, although EFM showed a reassuring fetal status 1 day before it occurred. Cord strangulation due to a detached amniotic membrane was found after stillbirth (Figure 1). Chorionic membrane separation (CMS) was observed at 32 weeks of gestation. There were 3 cases of CMS and 3 cases of pPROM out of 11 cases.

**Table 2:** Summary of adverse events in 11 cases in which FETO was performed

	*n* = 11
Procedure	
Maternal bleeding	0
Placental bleeding	0
Fetal injury	0
Pregnancy	
CMS	3 (27)
pPROM	3 (27)
Uterine infection	0
Placental abruption	0
Delivery <34 weeks of gestation	0
Survival	
Maternal death	0
Fetal death	1 (9)

Data are expressed as the number (%).

CMS, chorionic membrane separation; FETO, fetoscopic endoluminal tracheal occlusion; pPROM, preterm premature rupture of membranes.

Table 3 shows a summary of the 11 cases in which FETO was performed. The median o/e LHR at entry was 27% (range, 20–33%). The median gestational age at FETO was 30.9 weeks of gestation (range, 27.1–31.7 weeks). The median operation time was 33 min (range, 10–71 min). After FETO, the median o/e LHR at 33–34 weeks of gestation (before balloon removal) was 35% (range, 14–61%). In two cases, Kitano Grade 3 stomach position (>50% of the stomach herniated into the right chest) changed to Grade 2 (≤50% of the stomach herniated into the right chest) after FETO. The median gestational age at delivery was 36.5 weeks of gestation (range, 34.2–38.3 weeks). The median durations of occlusion (between balloon insertion and removal), that between balloon removal and delivery, and that between balloon insertion and delivery were 26 days (range: 17–49 days), 15 days (range 0–28 days) and 43 days (range, 21–66 days), respectively. CDH repair was performed after birth in 6 cases. The survival rate at 90 days of age and the rate of survival to discharge were both 45% (5/11).

**Table 3:** Summary of 11 cases in which FETO was performed

	*n* = 11
Characteristics	
Maternal age	31 (23, 40)
Primiparity	8 (64)
o/e LHR at entry (%)	27 (20, 33)
Liver up	11 (100)
Kitano G3 stomach position	11 (100)
Anterior placenta	5 (45)
Procedure	
Gestational age at FETO (weeks)	30.9 (27.1, 31.7)
Successful balloon insertion	11 (100)
Operation time (min)	33 (10, 71)
Gestational age at balloon removal (*n* = 10)	34.4 (34.0, 35.4)
Successful balloon removal (*n* = 10)	10 (100)
Balloon removal by EXIT	2 (20)
Findings after FETO at 33–34 weeks of gestation	
o/e LHR (%)	35 (14, 61)
Kitano G3 stomach position	9 (82)
Outcomes	
Gestational age at birth (weeks, *n* = 10)	36.5 (34.2, 38.3)
Duration of occlusion (days, *n* = 10)	26 (17, 49)
Duration between balloon removal and delivery (days, *n* = 10)	15 (0, 28)
Duration between balloon insertion and delivery (days, *n* = 10)	43 (21, 66)
Birth weight (g, *n* = 10)	2381 (2000, 2880)
Delivery by CS (*n* = 10)	7 (70)
CDH repair (*n* = 10)	6 (60)
Survival at 90 days	5 (45)
Survival to discharge	5 (45)

Data are expressed as the median (minimum, maximum) or number (%).

CDH, congenital diaphragmatic hernia; CS, caesarean section; EXIT, *ex utero* intrapartum treatment; FETO, fetoscopic endoluminal tracheal occlusion; G, grade; o/e LHR, observed/expected lung to head ratio.

## Discussion

In all 11 registered cases, FETO was successfully performed without severe maternal adverse events. In all 10 cases in which balloon removal was required, the balloon was successfully removed and the fetus was delivered at or after 34 weeks of gestation. This study demonstrated that it was feasible to perform FETO at an appropriately prepared fetal therapy center in Japan. This is the first successful FETO trial in Asia. FETO could be offered to women carrying fetuses with severe isolated left‐sided CDH to accelerate fetal lung growth.

The major obstacle to FETO is preterm delivery, which is the strongest determinant of mortality and morbidity in CDH.^15^, ^22^ Preterm delivery is associated with pPROM which is the most common complication of FETO.^15^ It was reported that pPROM occurred in 47% of 210 FETO cases and that the median gestational age at delivery was 35.3 weeks in a large cohort study.^15^ In recent small studies from other three groups, the incidence rates of pPROM were 30% in 10 cases,^17^ 71% in 21 cases^18^ and 61% in 28 cases,^19^ and the median gestational ages at delivery were 35.4 weeks,^17^ 34.7 weeks^18^ and 34.7 weeks,^19^ respectively. In our study, the incidence of pPROM was 27% in 11 cases and the median gestational age of delivery was 36.5 weeks. All 10 cases that were delivered at or after 34 weeks of gestation resulted in live birth.

The survival rate in the initial prospective study of FETO for severe CDH (liver herniation and LHR < 1) was 48% (10/21).^14^ The survival rate in a later large similar study of FETO for severe left‐sided CDH was 49% (86/175).^15^ In studies of FETO for severe CDH, the survival rates in a Brazilian randomized study, an Italian retrospective study and a Polish prospective study were 50% (10/20),^16^ 47% (8/17)^18^ and 46% (13/28),^19^ respectively. Although the survival in a small American study was 80% (8/10),^17^ all other studies have suggested that the survival rate among cases in which FETO is performed for severe CDH is around 50%. The survival rate in our study was 45%, which is comparable to previous published data from fetal treatment centers in other countries.

Observed/expected LHR and the liver position are the best‐studied prenatal prognostic predictors of CDH.^7^, ^23^, ^24^ Deprest *et al*. defined severe CDH as CDH with an o/e LHR < 25% in which the predicted survival rate was <20%, with cases without liver herniation showing better outcomes than those with liver herniation.^24^ The criteria that are commonly applied regarding the indication of FETO for severe CDH are left‐sided CDH with o/e LHR < 25% or LHR < 1.0, which is equivalent to o/e LHR < 25%.^14^, ^16^, ^17^, ^18^, ^19^ Some criteria also required liver herniation^14^.^17^ The severity of pulmonary hypoplasia in CDH is dynamic and the predictive value of the lung size in the second trimester is limited.^25^ The stomach position was proposed as a prognostic factor^26^, ^27^, ^28^, ^29^ and we proposed a new grading system for the stomach position that reflects the prognosis of CDH.^8^ Later, Cordier *et al*. also demonstrated the predictive value of the stomach position using a little different grading system.^30^ We included liver herniation and stomach position (Kitano Grade 3) in the entry criteria because the stomach position and o/e LHR were independent prognostic factors and the predicted intact survival discharge rate was <20% for patients with these factors.^8^

Unexpected fetal death after FETO was reported in 1 of 28 cases^19^ and 4 of 210 cases.^15^ The details of these five cases were not described and the etiologies were unclear. We had one unexpected fetal death case at 3 weeks after FETO. Umbilical cord strangulation caused by amniotic bands that formed following CMS was suspected as the cause of sudden fetal death. Similar cases have been reported after invasive procedures.^31^, ^32^ CMS is thought to be a potential lethal finding that can lead to cord strangulation and fetal compromise through amniotic bands.^31^ CMS occurred in 20% of patients after fetoscopic laser surgery for twin–twin transfusion syndrome and it was associated with pPROM but was not associated with survival.^33^ Although the incidence and the etiology of fetal death after FETO remain unclear, unexpected fetal death after FETO might occur and some cases associated with CMS may have resulted in umbilical cord compromise. Even in singleton pregnancies with spontaneous CMS, fetal death occurred in 6% of cases, with more than half of these deaths occurring due to cord strangulation.^34^ CMS after FETO should be recognized as a risk factor for not only pPROM but also for an umbilical cord accident leading to fetal death.

Regarding the timing of FETO, the median gestational age at FETO was 30.9 weeks and more than half of the cases in this study underwent FETO after 30 weeks of gestation. We intended to include cases of severe CDH by using liver herniation and Kitano Grade 3 stomach position as the entry criteria. However, we ultimately followed the method of an ongoing randomized controlled trial in which FETO was performed at 30–31 weeks of gestation for cases with an o/e LHR value of 25–44% to reduce the risk of preterm delivery^35^, ^36^ (www.totaltrial.ed). Although the performance of FETO prior to 29 weeks of gestation seems to be associated with a better lung response,^37^ the optimal timing and duration of tracheal occlusion, that balances the advantages for lung growth and the risk of preterm delivery, is still controversial. The median duration between FETO and delivery was 6 weeks and the gestational age at delivery was 1–2 weeks later in comparison to previous reports.^15^, ^17^, ^18^, ^19^ These results could be helpful when considering the timing of FETO.

The present study was associated with several strengths and limitations. The strength of this study was that the feasibility study was conducted for severe CDH cases characterized by Kitano Grade 3 stomach position, which predicted survival independently of o/e LHR. This is the only FETO study in which stomach position was an inclusion criterion. One limitation of this study was that FETO was performed in a relatively small number of cases and there was no expectantly managed control group. A large number of cases with well‐designed controls will be required to evaluate the effectiveness of FETO. An ongoing randomized controlled trial will draw conclusions regarding the usefulness of FETO^35^, ^36^ (www.totaltrial.ed). The second limitation of the present study was the timing of FETO. Cases with o/e LHR ≥25% underwent FETO at 30–31 weeks of gestation, although these cases were recognized as severe CDH due to Kitano Grade 3 stomach position.

In conclusion, FETO is feasible and was not associated with maternal morbidity in Japan, however, FETO is associated with a potential risk of fetal death in association with CMS. FETO could be offered to women carrying fetuses with severe isolated left‐sided CDH to accelerate fetal lung growth.

## Disclosure

None declared.
